# Reversals in Decisions About Life-Sustaining Treatment Among Terminally Ill Older Korean Patients

**DOI:** 10.1093/geroni/igaa057.794

**Published:** 2020-12-16

**Authors:** Suhyun Kim, Jung-Ja Choi

**Affiliations:** 1 Kyungpook National University, Daegu, Republic of Korea; 2 Kyungpook national university, Daegu, United States

## Abstract

Purpose: The purpose of this study was to investigate the patterns and factors of reversals in decisions about life –sustaining treatment (LST) among older patients with terminal stage of chronic cardiopulmonary diseases. Methods: In a retrospective correlational descriptive study, data were collected using medical chart review from 124 deceased older patients with terminal stage of cardiopulmonary disease who had made reversals of LST decisions in an academic tertiary hospital in 2015. Multivariate logistic regression analysis was used to identify the factors associated with the reversal to higher intensity of LST treatment. Results: Primary decision makers were offspring (72.6%), spouse (13.7%), acquaintance (8.9%), and patients (4.8%), in order. While 31.5% of the reversed decisions were made toward higher intensity of LST, 21.9% were made toward lower intensity of LST, and 46.6% were made for each treatment without change of overall code status. The use of inotropic was the most frequently reversed LST treatment (47.5%), followed by CPR (30.6%), intubation (27.4%), ventilator therapy (24.2%), and hemodialysis (17.8%). Patients who had lung diseases (vs. heart diseases), were single, divorced or bereaved (vs. married), and had acquaintance as a primary decision maker (vs. patients themselves) were significantly more likely to reverse the LST decisions to higher intensity of LST treatment. Conclusion: This study demonstrate the complex and turmoil situation of the LST decision making process among older patients with terminal stage of cardiopulmonary disease and suggests the importance of support for patients and families in their LST decision making process.

